# The acute effects of stress on dishonesty are moderated by individual differences in moral default

**DOI:** 10.1038/s41598-023-31056-2

**Published:** 2023-03-09

**Authors:** Sebastian P. H. Speer, Ana Martinovici, Ale Smidts, Maarten A. S. Boksem

**Affiliations:** 1 Rotterdam School of Management, Erasmus University, Rotterdam, The Netherlands; 2 Princeton Neuroscience Institute, Princeton University, Princeton, NJ USA

**Keywords:** Psychology, Human behaviour, Morality

## Abstract

In daily life we regularly must decide whether to act dishonestly for personal gain or to be honest and maintain a positive image of ourselves. While evidence suggests that acute stress influences moral decisions, it is unclear whether stress increases or decreases immoral behavior. Here, we hypothesize that stress, through its effects on cognitive control, has different effects on moral decision making for different individuals, depending on their moral default. We test this hypothesis by combining a task which allows for inconspicuously measuring spontaneous cheating with a well-established stress induction task. Our findings confirm our hypothesis, revealing that effects of stress on dishonesty are not uniform, but instead depend on the individual: for those who are relatively dishonest, stress increases dishonesty, whereas for participants who are relatively honest stress makes them more honest. These findings go a long way in resolving the conflicting findings in the literature on the effects of stress on moral decisions, suggesting that stress affects dishonesty differently for different individuals, depending on their moral default.

## Introduction

Our everyday life is full of situations in which we have to choose between behaving unethically for personal gain or to be honest and maintain a positive image of ourselves as being a ‘good person’. Just think of cheating on your tax returns, cheating in sports or board games or indeed fudging or faking research data. Often these moral conflicts need to be resolved under acute stress or can elicit stress themselves as for instance when considering cheating on an exam. Accumulating evidence suggests that acute stress influences moral decisions^1–6^. For instance, Starcke and colleagues^2^ found that a higher stress response was associated with more immoral decisions in emotional moral dilemmas. In contrast, von Dawans and colleagues^5^, found that stressed participants engaged in more moral behavior, as expressed by more trustworthy behavior and increased sharing. As illustrated by these examples, it is not at all clear whether stress increases or decreases immoral behavior.

A potential mechanism through which stress may influence moral decisions is cognitive control. Specifically, the Stress Induced Deliberation-to-Intuition (SIDI) model proposes that changes in decision-making under acute stress result from a combination of impaired cognitive control and enhanced intuitive response tendencies^7^. This model is supported by research showing stress-induced shifts from deliberative and goal-directed behavior to habitual and automatized behavior^8,9^. In addition, from a neural perspective, it has been found that stress impairs prefrontal functions such as directing attention and inhibiting inappropriate actions, which are essential components of self-control^10,11^. More precisely, it has been shown that stress is associated with reduced functional connectivity between the dorsolateral prefrontal cortex (dlPFC) and the ventromedial prefrontal cortex (vmPFC)^12^. A strong functional connectivity between these two brain areas has been associated with successful self-control^13,14^. Moreover, higher cortisol levels, associated with acute stress, were found to be correlated with increased connectivity between vmPFC, ventral striatum and amygdala, which the authors interpret as an enhanced motivational signal favoring the immediately rewarding option^12^. Taken together, in accordance with the SIDI model^7^, these findings suggest that stress biases the decision process in the brain by reducing self-control capacities while increasing the motivational salience of intuitive actions that are often associated with immediate reward.

In a series of recent studies it was shown that this capacity for cognitive control, in particular the inhibition of predominant responses, also plays an important role in dishonesty^15^. These studies reveal that there are substantial individual differences in (dis)honesty^16–19^, with some being extremely honest and some being completely dishonest, and everything in between. These individual differences to a large extent determine how individuals employ cognitive control to resolve moral conflict. It was found that cognitive control is not needed to be honest or dishonest per se, but that it depends on an individual’s moral default. Cognitive control, in the form of inhibition of intuitive responses, allows individuals to override their moral default, helping intuitively dishonest participants to be honest, whereas it allows for cheating in intuitively honest participants^17,18^.

In light of these findings, we hypothesize that stress, through its effects on cognitive control, will have different effects on moral decision making depending on a person’s moral default. Specifically, we hypothesize that a person who is intuitively honest will be less able to override their moral default and will therefore cheat less under stress. In contrast, for cheaters we hypothesize that they will cheat more after being exposed to stress, as self-control processes are impaired and intuitive decisions are facilitated. We thus expect an interaction effect between stress and a person’s moral default: A person’s moral default determines whether stress increases or decreases honesty. Since the size of the overall effect of stress on cheating is contingent on the relative number of cheaters vs. honest participants in the sample, we do not hypothesize the direction of any main effect of stress.

In the current study we test the hypothesis that a person’s moral default moderates the effect of stress on (dis)honesty by combining the newly developed Spot-The-Differences Task (SPOT)^18^, which allows for inconspicuously measuring spontaneous cheating on a trial-by-trial basis, with the Maastricht Acute Stress Test (MAST)^20^, a well-established stress induction task. We implemented a mixed design in which participants completed a first block of the SPOT cheating task, were then exposed to either a psychosocial stress task or a matched control task, and then ended the experiment with the second and final block of the SPOT. An individual’s level of cheating in the first block of the SPOT is taken as a measure of the participant’s moral default. This design allowed us to test for differences in cheating behavior due to the induction of acute stress, while accounting for participants’ moral default.

## Method

### Participants

We recruited 120 participants (62 females; age 18–28 years; M = 20.6, SD = 2.28) from an online community for university students, where students can sign up for experiments. All participants provided written informed consent for their participation. The study was approved by the Erasmus Research Institute of Management (ERIM) internal review board and was conducted according to the Declaration of Helsinki.

### Design

Participants completed two tasks in this study: two blocks of the Spot-The-Differences task (SPOT)^18^ to measure cheating behavior and the Maastricht Acute Stress Test (MAST)^20^, in between the two blocks of the SPOT task to induce acute stress. Random assignment of participants to one of two conditions (acute stress and control) allowed us to test the effect of acute stress on cheating by comparing differences in cheating behavior in the acute stress versus control group.

### SPOT

**Figure 1:**
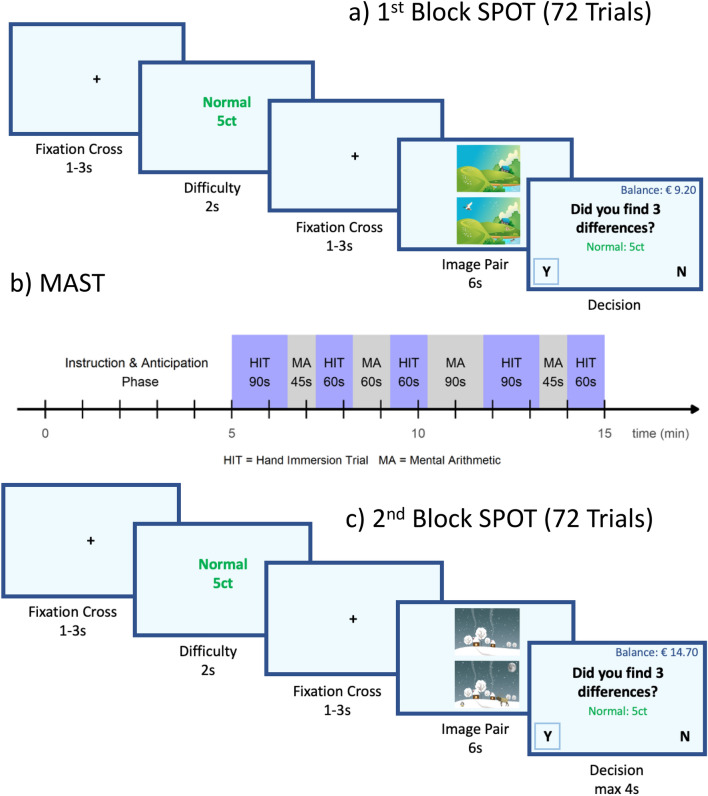
Overview of the experimental procedure. Participants first completed the first half (72 trials, of which an average of 33 were cheatable trials of interest) of the SPOT. On each trial participants viewed a screen indicating the difficulty and reward for that trial, then the image pair appeared for 6 s and finally participants had to indicate whether they spotted all 3 differences. Subsequently participants completed the MAST in which the HIT and MA task were alternated with the order and timing presented here. *HIT* hand immersion task, *MA* mental arithmetic (reproduced based on Speer et al., 2022b; Smeets et al., 2012. SPOT images are downloaded from Pixabay, https://pixabay.com/service/license/).

In the SPOT participants were presented with pairs of cartoon images (see online Appendix 1 for validation of the images) and instructed that there are always three differences between the images and their task is to find them (see Fig. 1a,c for a timeline of an example trial). Differences consisted of objects that were added to or removed from an image, or objects that differed in color between images https://osf.io/bcm7u/ for example images). Participants were instructed that the purpose of the study was to investigate the accuracy of visual search for marketing purposes such as searching for a product in an assortment or information on a webpage. In order to increase credibility of this cover story, a simple visual search task was added at the beginning of the experiment (see online Appendix 2). Further, participants were instructed that the effect of motivation, elicited by monetary reward, on speed and accuracy of visual search would be investigated. The monetary reward for finding the three differences between the images was determined by the stated difficulty of each trial: 5 cents for ‘normal’, 20 cents for ‘hard’, and 40 cents for ‘very hard’ trials.

Crucially, although participants were told that there were three differences in all trials, in 25% of the trials there were only two differences and in 25% there was only one difference. Since reward (see below) was contingent on participants reporting that they had found all three differences, without having to point them out, this design allowed and encouraged cheating behavior (i.e., reporting having found all three, even when objectively fewer than three differences were present in the images).

Trials were categorized as normal (44.7%), hard (30.7%) and very hard trials (24.6%), for which participants could receive 5cts, 20cts, and 40cts, respectively. The majority of trials with three differences (the filler trials) were categorized as normal trials (88%) and the majority of trials with one or two differences (cheatable trials) were categorized as hard or very hard (98%, the trials of interest used in the analysis). To reduce suspicion that none of the harder trials actually contain three differences, some of the three difference trials were categorized as hard or very hard and some of the trials with one or two differences were categorized as normal. The reward was independent of the number of differences in the image pair for the trials of interest. The different levels of difficulty were added to reduce suspicion about the real purpose of the task. It was assumed that if trials are labeled as hard or very hard, it would be more credible to the participant that the image pair actually contained three differences, but they were just too hard to spot. In addition, levels of difficulty were introduced to eliminate possible demand effects: we wanted participants to cheat for monetary reward and not to prevent seeming incompetent, which may be associated with different underlying psychological mechanisms and consequently confound the analysis.

To further reduce suspicion about the purpose of the study, approximately 10% of all trials (12 trials) were point-and-click trials. In these trials, participants had to click on the location in the images where they spotted the differences, and they could only click three times, after which the trial advanced. If participants clicked on the wrong locations, they did not receive the reward for that trial. Participants always knew prior to the start of a trial if it was a point-and-click trial, indicated by a screen requesting participants to click on the image. They were told that only approximately 10% of trials were point-and-click trials because it would take too much time to point out the differences for every pair. For the point-and-click trials 12 additional stimuli were used, all of which had three differences between the images, six labeled as normal, three as hard and three as very hard.

The maximum amount of money earned if a participant cheated on all cheatable trials was approximately 25 Euros, whereas if a participant did not cheat at all, he or she would earn approximately 4 Euros. After completion of the full study, participants were debriefed. Then, all participants received the maximum of 35 Euros (including the 10 Euros show-up fee) irrespective of their actual cheating behavior.

### MAST

The MAST is a well-established stress paradigm which is used in various scientific disciplines ranging from cognitive and social neuroscience to psychology and medical sciences^21–23^. The MAST combines social with physical stressors and has been found to reliably induce elevated cortisol levels and subjective ratings of stress. The MAST consists of a 5 min preparation phase and a 10 min acute stress phase (in total 15 min). It is a combination of a Hand Immersion Task (in ice cold water, see below) which induces physical discomfort and a socially evaluative aspect, involving being judged on a Mental Arithmetic Task and being filmed throughout the whole task, which induces social stress. Hand Immersion and Mental Arithmetic trials are presented alternatingly and with varying lengths (see Fig. 1b). It is important to note that while participants were under the impression of being filmed by means of a visible camera set up, no actual recordings were made for privacy reasons. The MAST control condition consists of immersing a hand in lukewarm water and conducting a far simpler mental arithmetic task without social evaluation.

Figure 1b presents an overview of the MAST procedure. The 5 min preparation period served to seat participants in front of a computer screen and instruct them about the upcoming task via a PowerPoint presentation. Participants were informed about the Hand Immersion task, the Mental Arithmetic Task, and that they were going to be monitored by the experimenter as well as videotaped so as to later analyze their facial expressions.

For the Hand Immersion Task, participants were instructed to immerse their hand up to and including the wrist in ice-cold water (temperature at the start of the session (degrees Celsius): M = 2.24, SD = 0.55; temperature at the end of the session: M = 2.92; SD = 0.7) while they were being videotaped and closely monitored by an experimenter who displayed a lack of empathy, by keeping a neutral facial expression in spite of the participants’ discomfort. It was made clear that participants were allowed to withdraw their hand at any time (n = 6 participants made use of this option; results in online Appendix 3 show that the main findings are robust to exclusion of these participants).

The Mental Arithmetic Task consisted of counting backwards starting at 2043 in steps of 17 as fast and accurately as possible. Also during this mental arithmetic task, participants were led to believe that they were videotaped. Each time they made a mistake, they were given negative feedback (the experimenter verbally pointed out they made a mistake) and were requested to start over at 2043.

The control condition was identical to the experimental condition, except that there was no camera present, participants were told that the water was lukewarm (temperature at the start of the session (degrees Celsius): M = 35.21, SD = 1.69; temperature at the end of the session: M = 32.27; SD = 1.75), and the arithmetic task consisted of counting from 1 to 25 at their own pace starting anew at 1 when 25 was reached. The experimenter remained in the room to check participants’ compliance with the instructions, but participants were not given any feedback on their performance.

### Procedure

To explore the effects of acute stress on (dis)honest behavior, a mixed pretest–posttest design was implemented, in which participants made (dis)honest decisions in the two blocks of the SPOT: one block before the MAST or the associated control condition and one block after. The effect of acute stress induced by psychosocial stress paradigms was estimated to last around 15 min^24^ with elevated cortisol levels for the MAST still detectable 40 min after beginning of the MAST^20^. Each block of the SPOT had a duration of approximately 15 min on average, so performance in the second block fell well within the window of expected elevated stress levels. Participants first completed the first block of the SPOT (15 min for one block; 72 trials). Subsequently, participants were randomly assigned to complete either the MAST or the control condition (15 min). Finally, participants finished the second block of the SPOT (15 min). This design allowed us to compare the effect of stress on (dis)honest decisions. At the end of the experiment participants completed a short questionnaire testing whether participants were suspicious about the real purpose of the task (5 min).

### Logistic regression model of cheating behavior

The probability that participant $i$ cheats in trial $j$ (${p}_{ij}({cheat}_{ij}=1)$) during the second block of the SPOT is ${p}_{ij}=\frac{exp({Y}_{ij})}{exp({Y}_{ij})+1}$, where:

$${Y}_{ij}={\beta }_{0 }+{\beta }_{1 }* BaselineCheatin{g}_{i }+{\beta }_{2 }* Stres{s}_{i}+{\beta }_{3 }* BaselineCheatin{g}_{i }* Stres{s}_{i}$$

The intercept (${\beta }_{0}$) captures the overall tendency to cheat. The parameter ${\beta }_{1}$ accounts for moral-default effects on the probability to cheat. Specifically, the extent to which participants cheated during the first block of the SPOT ($BaselineCheatin{g}_{i}$) is expected to impact cheating behavior in the second block of the SPOT. $BaselineCheatin{g}_{i}$ is a standardized measure (i.e., z-score) of the percent of cheating in trials of interest during the first block of the SPOT. The ${\beta }_{2}$ parameter corresponds to the main effect of acute stress. $Stres{s}_{i}$ is a dummy variable that indicates if participant $i$ is randomly assigned to the experimental ($Stres{s}_{i}=1$) or control ($Stres{s}_{i}=0$) condition of the MAST. The parameter ${\beta }_{3}$ captures the hypothesized interaction between cheating in the first block of the SPOT and acute stress.

We use Bayesian methods to estimate model parameters with RStan (Version 2.26.13)^25^, R^26^, and RStudio^27^. We used 10 chains, each with 10,000 iterations (first 5000 used for warmup). Convergence was indicated by Rhat measures equal to 1 for all parameters. We use the 50,000 posterior draws to calculate, for each parameter (${\beta }_{0}$– ${\beta }_{4}$): the mean, 95% credible interval (CI) bounds, and Bayesian *p *value (smallest mass of the posterior distribution not including zero). The credible interval is the range of the posterior distribution that contains a percentage of probable values. Therefore, the 95% credible interval is the central portion of the posterior distribution that contains 95% of the values.

## Results

**Figure 2:**
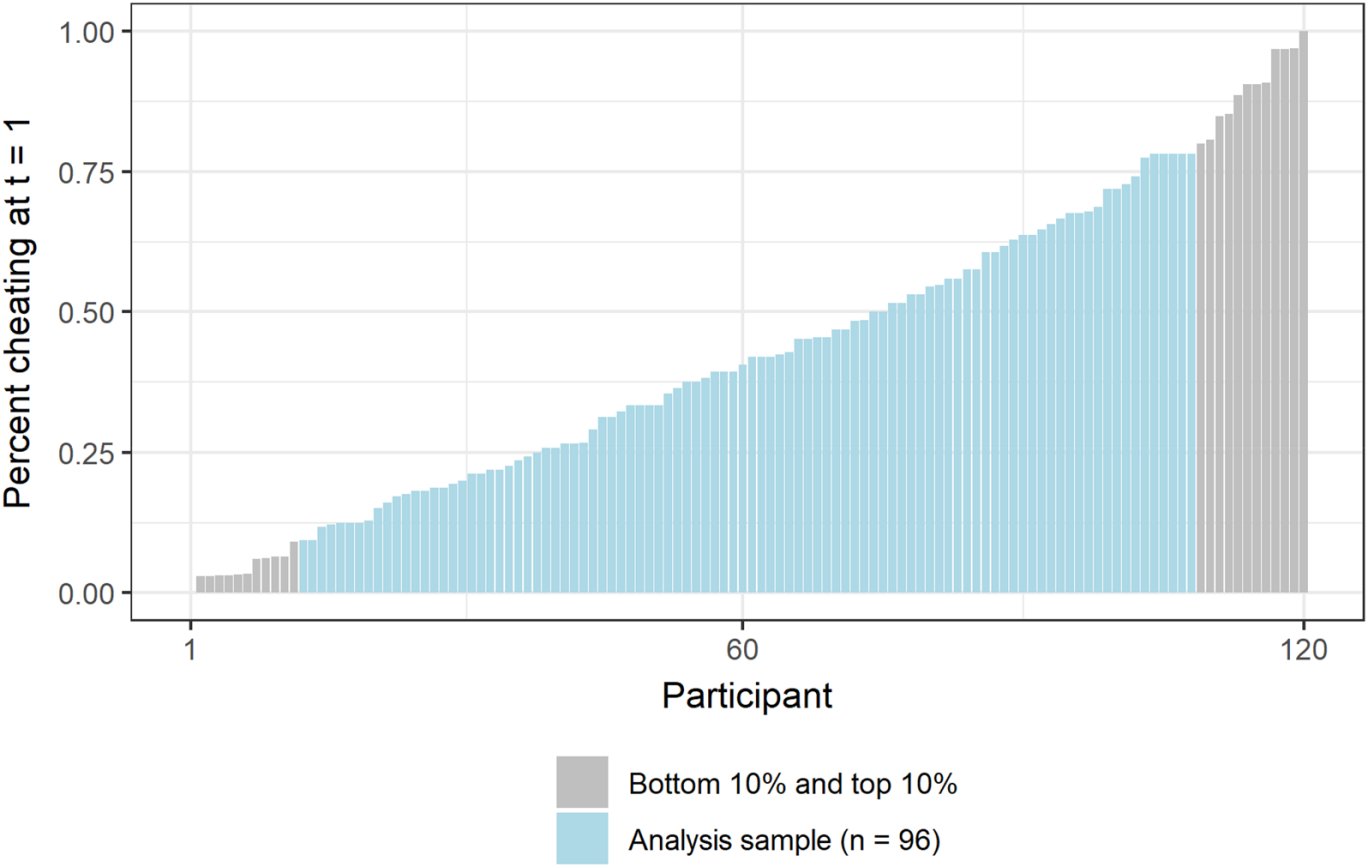
Observed cheating behavior in the first block of the SPOT. Participants are sorted based on the percent of trials they cheated, with the 10% most honest participants on the left side of the figure and the 10% most dishonest on the right side of the figure. On average, the 10% most honest participants cheated on 4% of trials (on average 1 trial out of 32), while the 10% most dishonest participants cheated on 90% of the trials (on average 29 out of 32 trials).

There was substantial variability in how much participants cheated in the first block of the SPOT. On average participants cheated on 43% of the trials of interest (SD = 0.27), but the most honest participant did not cheat on any of the trials, while the most dishonest participant cheated in all trials (Fig. 2). Obviously, the extent to which participants can behave even more strongly in line with their moral default due to acute stress is limited by their behavior during the first block of the task. For example, the participant who was always honest at t = 1 cannot become even more honest (floor effect) as a result of the stress treatment, while the participant who cheated in all of the trials at t = 1 cannot cheat even more after stress (ceiling effect). To eliminate ceiling and floor effects, we performed the analysis on a subsample of participants (n = 96) who have a standardized cheating measure between the 10th and 90th percentiles. As a robustness check, we show that model estimation results for the subsample are very similar to those obtained with the full sample (see Appendix 3).

Importantly, no statistically significant difference was found between the control and stress groups in the percent of trials participants cheated during the first block of the SPOT (*F*(1, 94) = 1.15, *p *value = 0.29). The mean (SD) cheating in the first block is 0.44 (0.20) for the control group (n = 52) and 0.39 (0.20) for the stress group (n = 44). In the second block, the mean (SD) is 0.43 (0.29) for the control group and 0.43 (0.31) for the stress group.

We analyzed the effect of acute stress on cheating behavior by using a logistic regression model estimated on behavior in the second block on the SPOT. The probability that a participant cheats during the second block of the SPOT is influenced by: (1) their moral default (as operationalized by the extent to which the participant cheated during the first block of the SPOT), (2) stress (indicating whether the participant was assigned to the experimental condition of the MAST or the control condition), and the interaction between (1) and (2).

**Figure 3:**
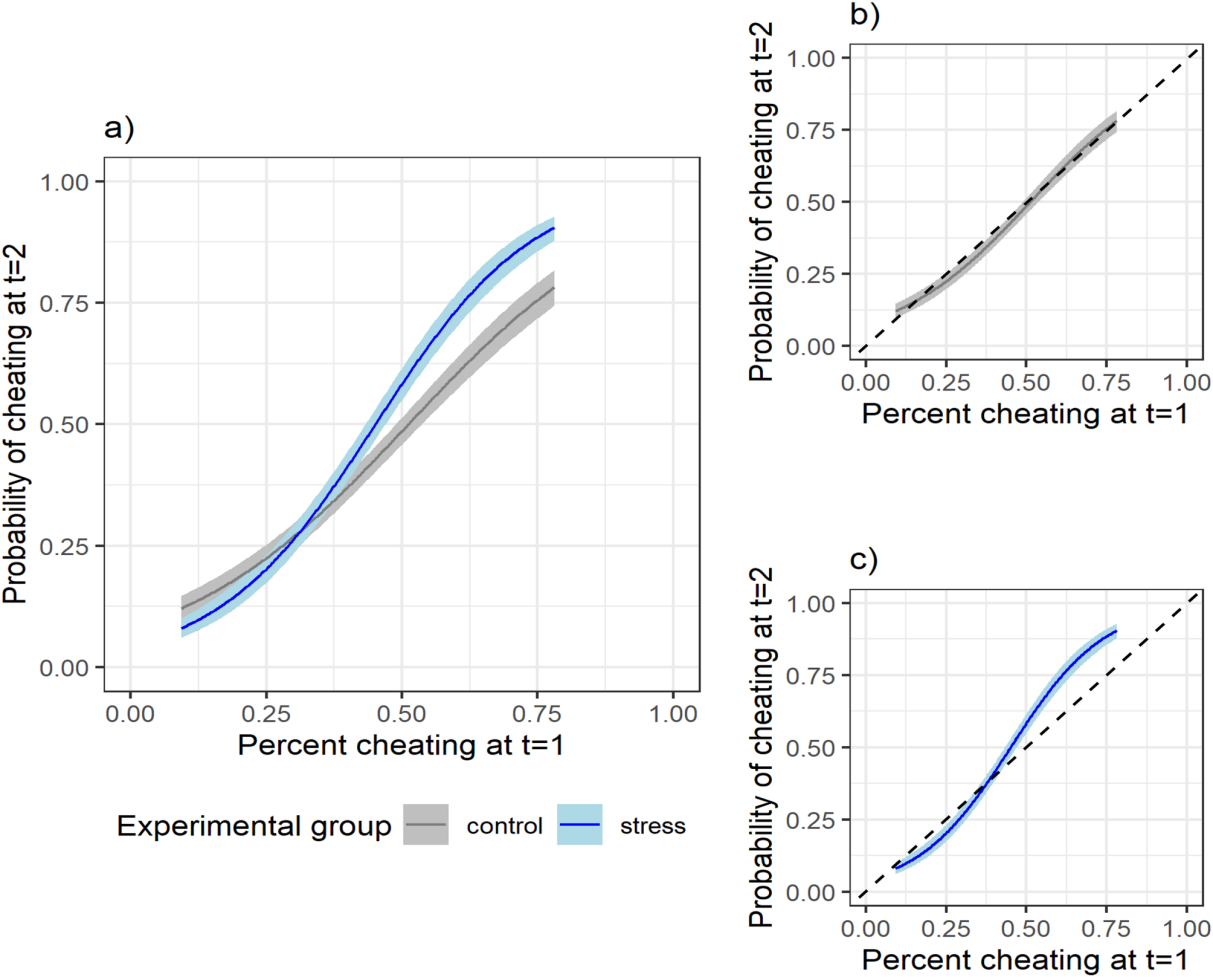
Estimated probability of cheating in the second block of the SPOT (t = 2) as a function of cheating during the first block (t = 1) and experimental group (n = 96 participants, with n = 52 in the control and n = 44 in the stress group), combined (panel **a**) and split for control (**b**) and stress group (**c**). The range of the x-axis matches the observed variation in cheating. Confidence bands represent 95% credible intervals (CI).

We found that cheating in the first block of the SPOT (t = 1) has a positive effect on the probability of cheating in the second block (t = 2) (estimated mean equal to 1.26, 95% credible interval (CI) of [1.11; 1.41], and *p *value < 0.001). This shows that the level of cheating between the two blocks is strongly correlated, with participants who cheated more at t = 1 being also more likely to cheat at t = 2. In addition, the results showed a statistically significant effect of stress on the probability of cheating at t = 2 (estimated mean equal to 0.23, 95% CI = [0.07; 0.40], and *p *value = 0.003). Most importantly, we found support for an interaction effect between stress and moral default (estimated mean equal to 0.52, 95% CI = [0.28; 0.77], and *p *value < 0.001). To show the combined effect, Fig. 3a presents the estimated probability of cheating in the second block of the SPOT (t = 2) for participants in the control and stress group for varying levels of cheating during the first block (t = 1). The results show that participants in the stress group who cheat often in the first block have a higher probability of cheating at t = 2 compared with similar participants in the control group. Conversely, very honest participants tend to become more honest after stress, although this effect is less pronounced. Figure 3b (control group) and c (stress group) further illustrate the effect of stress in interaction with the moral default, by showing the probability of cheating relative to the diagonal, which represents no change in cheating between the first and second block. Indeed, for the control group, no change in cheating is observed (Fig. 3b). Instead, and as hypothesized, Fig. 3c clearly shows that: (1) honest participants in the stress group have a lower probability of cheating in the second block compared to the first block, whereas (2) dishonest participants in the stress group have a higher probability of cheating in the second block compared to the first block.

## Discussion

In this study we investigated the effect of acute stress on (dis)honesty. We found that effects of stress on dishonesty are not uniform, but that they depend on an individual’s moral default: for those who are relatively dishonest, stress increases dishonesty, whereas for more honestly inclined participants, stress makes them even more honest.

To assess participants’ default inclination to cheat (i.e., their tendency to cheat in the absence of stress) participants completed a first block of the SPOT, a task designed to measure trial-by-trial cheating inconspicuously. Participants were then exposed to the MAST (or its control condition) to induce acute physical and social stress. Examining behavior during the second block while accounting for behavior in the first block enabled us to assess the effects (main effect and interaction) of an individual’s moral default and acute stress on cheating.

We found that for most participants stress increased dishonesty. Most importantly, however, when accounting for individual differences in tendency to cheat (as reflected by cheating in the first block of the SPOT), a significant interaction effect was observed. Our model revealed that generally dishonest participants became more dishonest under acute stress as compared to the control condition (no stress), whereas this was not the case for more honest participants. When comparing participants’ behavior after stress to their own behavior before stress (at baseline) a similar pattern emerged: dishonesty increased for the dishonestly inclined, and honest participants became more honest as compared to baseline. Thus, the results support our hypothesis that acute stress enhances participants’ default tendency for (dis)honesty, i.e., their *moral default *^15^.

As mentioned in the introduction, the SIDI model^7^ proposes that acute stress influences decisions by impairing cognitive control and promoting intuitive response tendencies. Neuroimaging research on dishonesty has shown that people differ in their moral defaults and that people require cognitive control to override these defaults: individuals who are by default honest need willpower, in the form of cognitive control, to cheat, whereas cheaters need cognitive control to be honest ^15^. This line of research has shown that a network of brain regions associated with cognitive control is more strongly activated when honest people cheat, whereas for cheaters the same network is more engaged when they decide to be honest^17,18^. It follows that if cognitive control is impaired by acute stress this should impact dishonesty differently for different people. Taken together, our current findings, the literature on the impact of stress on cognitive control, and the neuroimaging literature on dishonesty suggest that acute stress impairs cognitive control and would thus leave individuals less able to override their moral default. As a result, lacking sufficient cognitive control resources under stress, participants with an honest default consequently rely more on their intuition to be honest, while cheaters under stress will be more likely to succumb to their default dishonest tendencies.

The moderating role of the moral default on the effect of stress on dishonesty may help reconcile the mixed results in the literature on stress and moral behavior. For example, while Starcke and colleagues^2^ found that stress decreased moral behavior, von Dawans and colleagues^5^ observed an increase in moral behavior after stress. The current findings suggest that both patterns of results are plausible, depending on the composition of the sample in terms of individual moral defaults.

Similar mixed findings have been observed in the context of prosocial behavior. A recent review by Faber and Häusser^1^ reported that results from studies investigating the effect of stress on prosocial behavior ranged from increased prosociality, to no behavioral differences and mixed effects contingent on moderators, all the way to decreased prosocial behaviour. Similarly, Nitschke and colleagues^28^ reported an absence of a reliable effect of acute stress on prosocial behavior across various economic games.

While being honest is not necessarily the same as being prosocial, there is significant overlap between the two concepts. The main difference here is that in our study there is no identifiable victim of any moral transgression, so the observed dishonesty cannot necessarily be called antisocial. Nevertheless, honesty and prosocial behavior typically go hand in hand, so the current findings may inform the literature on prosocial behavior as well. Specifically, inconsistencies in that literature may also have come about because a priori individual differences in prosociality have not been taken into account. Accounting for these individual differences may help resolve the controversy on the effect of stress on prosocial decision making. In line with this reasoning, a recent study by Forbes and colleagues^29^ found that individuals with a more selfish social value orientation exhibit even more selfish behavior under acute stress, whereas this was not observed for individuals with a more prosocial value orientation. Moreover, Azulay and colleagues^30^ reported that individuals with high trait empathy became more prosocial after acute stress, whereas individuals with low trait empathy became more selfish. In concert, these findings suggest that heterogeneity in prosociality, as represented by differences in trait empathy levels and social value orientation, moderate the effect of acute stress on prosocial decisions (but see^31^.

Because some participants cheated (nearly) all the time and some participants (almost) never, ceiling and floor effects were to be expected. Specifically, if a participant cheated very often in the first half of the study there was hardly any possibility for stress to further increase cheating in the second half. Cheating by one trial more (e.g., increase cheating from 29 to 30 of 32 trials), albeit possible, corresponds to a very small effect size that requires a much larger number of observations to be detected. Similarly, for extremely honest participants who cheat in only one of the trials during the first half of the experiment there is very little room to become more honest. Therefore, we analyzed the effect of stress and moral default after removing the 10% most extreme participants from the sample. Importantly, we also included a sensitivity check in which all participants were included, even those whose behavior can’t be further changed, and also analyses in which we removed the top and bottom 5% and 15% of participants (see Appendix 3). These analyses revealed that the interaction effect remains significant even with all participants included or with different exclusion criteria. However, whereas the main analysis (excluding the most extreme 10% participants) showed a large stress-induced increase in cheating for cheaters and a smaller decrease in cheating for honest participants, including all participants mainly revealed an effect of stress on cheaters (i.e., increasing cheating). Thus, when allowing stress to have more impact on behavior (by removing those who can’t be influenced), we observed that stress indeed potentiates participants’ default (dis)honesty on both ends of the moral default distribution. Nevertheless, the effect of stress on dishonesty seems most pronounced for those with a relatively dishonest moral default.

A potential explanation for the finding that effects of stress on dishonesty are not only not uniform, but also not entirely symmetrical (i.e., the impact of stress on honesty for those with an honest default appears less pronounced as compared to those with a dishonest default), may lie in the fact that stress not only impacts on cognitive control, but also on mentalizing^31–33^. FMRI studies have found that a network of brain areas associated with mentalizing, such as the posterior cingulate cortex, temporo-parietal junctions, and the medial prefrontal cortex^34^, was more strongly activated and more intrinsically connected for honest participants being honest^15,18^.Taken together, this may explain our finding that the effect of stress on honest individuals seems smaller than on those who are dishonest by default: for honest people, stress reduces cognitive control (making them more honest) but also reduces mentalizing (making them less honest). So, stress has two opposing impacts on honesty for honest people, resulting in just a small increase in honesty after stress. For dishonest individuals a reduction in control and a reduction in mentalizing both make them more dishonest, resulting in the relatively large increase in dishonesty. More research is called for to explicitly test this hypothesis.

One limitation of this study pertains to the fact that it was not tested whether the MAST indeed induced stress in the participants. While the MAST is a well-established stress paradigm which has been used in various scientific disciplines and has been found to reliably induce elevated cortisol levels and subjective ratings of stress^21–23^, more certainty about successful stress induction could have been derived from cortisol samples or physiological measures such as heart rate or skin conductance.

It should also be noted that participants who were more likely to cheat or be honest under stress in this specific experimental paradigm may not show the same behavior under acute stress in a different paradigm designed to elicit (dis)honesty. Nevertheless, previous research has shown that cheating on the SPOT task is associated with stable neural patterns in the brain as well as well-established personality measures associated with impulsivity^19^, which suggests that the individual differences in cheating observed in our paradigm may indeed reflect stable variations in moral default. More research is needed, however, to evaluate the generalizability of the role of the moral default on the effect of stress on (dis)honesty in other experimental paradigms and contexts.

Lastly, we can now only speculate that stress influences (dis)honesty by interfering with cognitive control processes, as cognitive control was not explicitly measured. A promising way to test this direct causal link empirically would be to combine the experimental design used in this study with transcranial magnetic stimulation (TMS). TMS a noninvasive brain stimulation technique in which an alternating magnetic field is used to create an electric current at a localized region of the brain through electromagnetic induction. It can be used to upregulate or downregulate certain areas of the brain and is therefore useful to test the causal involvement of these areas in a given neurocognitive process. By downregulating (or upregulating) brain regions associated with cognitive control during SPOT (after acute stress) researchers could measure the causal involvement of cognitive control in the effect of stress on (dis)honesty.

To conclude, our findings show that the effects of acute stress on dishonesty are not uniform but are contingent on an individual’s moral default. While stress increased cheating for the dishonestly inclined, more honest participants did not cheat more under stress and even became slightly more honest. These findings go a long way in resolving the conflicting findings in the literature on the effects of stress on dishonest behavior, with some studies reporting an increase and others a decrease in ethical behavior. The current findings suggest that stress affects ethical behavior differently for different people, depending on their moral default.

## Supplementary Information

Supplementary Information.

## Data Availability

Data and code to reproduce the analysis in the paper are available at: https://github.com/anamartinovici/stress_and_honesty. The GitHub repository is linked to an OSF project (https://osf.io/bcm7u/) with https://doi.org/10.17605/OSF.IO/BCM7U.
