# Supplementary material for: The acute effects of stress on dishonesty are moderated by individual differences in moral default
Source: Sci Rep. 2023 Mar 9;13:3984. doi: 10.1038/s41598-023-31056-2 (PMC9998439; doi:10.1038/s41598-023-31056-2)
Supplement: Supplementary file 1 — Supplementary Information. [file 41598_2023_31056_MOESM1_ESM.docx]

**Appendices**

**Appendix 1: Validation of the SPOT picture set**

Stimuli for the task consisted of 144 Spot-The-Differences image pairs that were downloaded from the Internet. Cartoon images of landscapes containing several objects were selected to make them engaging and challenging enough for the participants. Landscapes were chosen as they generally satisfied the necessary criteria of containing several different objects, which made the task of spotting differences more challenging and engaging. The stimuli consist of pairs of images that are identical apart from a certain number (1-3) of differences that were created by the experimenter using Adobe Photoshop. Differences consisted of objects added to or removed from the landscape picture or changed colors of objects.

To make sure that participants would be able to find the differences between the images in a reasonable amount of time, we ran a pilot study on Amazon’s Mechanical Turk with 205 subjects using 180 pictures to test the difficulty to spot the differences between the images and to determine the optimal duration of picture presentation. Participants were presented with cartoon image pairs, presented horizontally next to each other, containing three differences, and were asked to click on the differences identified in the image on the right-hand side. They were given 15 seconds to make their response. Using the heatmap function provided by Qualtrics, regions of interest were defined around the locations of the differences in the image on the right-hand side and response times for each of the clicks were recorded. This allowed us to test whether participants were able to find all differences in an image pair, which differences were particularly difficult to find, and how long it took to identify all differences. Based on the responses of these 205 participants, 36 image pairs that took too long or had differences that were too difficult or too easy, were removed, resulting in 144 images that took 92% participants less than 6s to find all three differences (M=5.4s, SD =1.5s).

To get a better sense of how difficult it was to spot the difference between images if there were indeed three differences, example images were uploaded to OSF (https://osf.io/bcm7u/).

**Appendix 2: Visual search task**

To further increase the credibility of our cover story on brain processes underlying visual search, we also included the visual search task introduced by Treisman and Gelade (1980) at the beginning of our experiment. Specifically, participants were told that the experiment would start with a simple visual task and then proceed to visual searches in more complex visual stimuli in the second task. In this first task, the goal was to determine whether a specific target was present or absent. In each trial participants were presented with colored letters presented in random locations on the screen. If the target was present, then participants had to press the left mouse button as quickly as possible. If no target was present, then they had to press the right mouse button as quickly as possible. For this task, participants had to search for a green T. Participants were instructed to answer as quickly as possible while still being as accurate as possible. The task took approximately 5 minutes and was not analyzed as it was included solely for the purpose of increasing the credibility of our cover story.


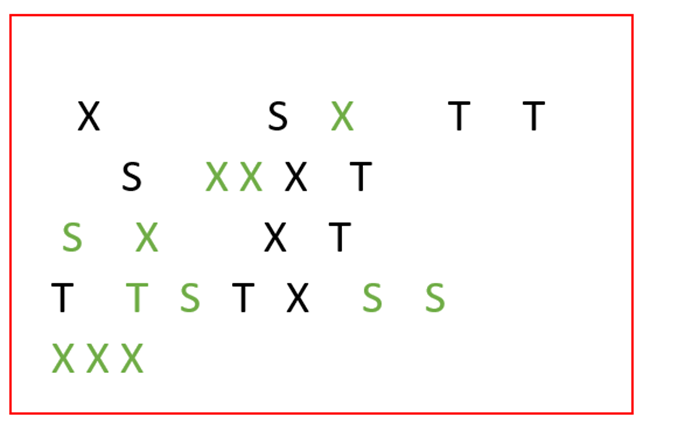


Figure 1. One trial of the simple visual search task. Participants have to indicate whether a green T is among the letters on the screen.

**Appendix 3: Results sensitivity checks**

The probability that participant $i$ cheats in trial $j$ ($p_{ij}({cheat}_{ij}=1)$) during the second block of the SPOT is $p_{ij}=\frac{exp(Y_{ij})}{exp(Y_{ij})+1}$, where:

$$Y_{ij}=\beta_{0}+\beta_{1}* BaselineCheating_{i}+\beta_{2}* Stress_{i}+\beta_{3}* BaselineCheating_{i}* Stress_{i}$$

The intercept ($\beta_{0}$) captures the overall tendency to cheat. The parameter $\beta_{1}$ accounts for moral-default effects on the probability to cheat. Specifically, the extent to which participants cheated during the first block of the SPOT ($BaselineCheating_{i}$) is expected to impact cheating behavior in the second block of the SPOT. $BaselineCheating_{i}$ is a standardized measure (i.e., z-score) of the percent of cheating in trials of interest during the first block of the SPOT. The $\beta_{2}$parameter corresponds to the main effect of acute stress. $Stress_{i}$ is a dummy variable that indicates if participant $i$ is randomly assigned to the experimental ($Stress_{i}=1$) or control ($Stress_{i}=0$) condition of the MAST. The parameter $\beta_{3}$ captures the hypothesized interaction between cheating in the first block of the SPOT and acute stress.

We use Bayesian methods to estimate model parameters with RStan (Version 2.26.13, Stan Development Team 2022), R (R Core Team 2022), and RStudio (RStudio Team 2022). We used 10 chains, each with 10000 iterations (first 5000 used for warmup). Convergence was indicated by Rhat measures equal to 1 for all parameters. We use the 50000 posterior draws to calculate, for each parameter ($\beta_{0}$- $\beta_{4}$): the mean, 95% credible interval (CI) bounds, and Bayesian *p*-value (smallest mass of the posterior distribution not including zero).

Results in the paper are based on a sample of n = 96 participants that excludes the top 10% and bottom 10% participants based on the standardized measure of cheating in the first block of the Spot-The-Differences task. We systematically checked how sensitive the results are to different exclusion criteria. This appendix reports four additional sets of results based on: the full sample (n = 120), a subsample of n = 109 participants that excludes the top 5% and the bottom 5% participants based on the standardized measure of cheating in the first block of the SPOT, a subsample of n = 86 participants that excludes the top 15% and bottom 15% participants, and a subsample of n = 114 participants that excludes the six participants who withdrew their hand at least once during the hand immersion task. The results are very similar to those reported in the paper, which shows that there is very little, if any, sensitivity to different ways of determining very honest (bottom %) and very dishonest (top %) participants, and to compliance during the hand immersion task.

Results with the full sample (n = 120)

Cheating in the first block of the Spot-The-Differences task has a positive effect on the probability of cheating in the second block (estimated mean = 1.36, 95% CI = [1.23; 1.50], p-value < .001). The results show a statistically significant effect of the Stress treatment on the probability of cheating (estimated mean = .20, 95% CI = [.05; .36], p-value = .005). In addition, we find support for an interaction effect between stress and baseline cheating (estimated mean = .18, 95% CI = [-.01; .37], p-value = .028). No statistically significant difference was found between the control and stress groups in the percent of trials participants cheated during the first block of the SPOT (*F*(1, 118) = .01, *p*-value = .91). The mean (SD) cheating for the control group (n = 60) is .43 (.25) in the first block and .42 (.31) in the second block. For the stress group (n = 60), the mean (SD) cheating is .43 (.28) in the first block and .45 (.34) in the second block.

Results with a subsample (n = 109) that excludes the bottom 5% and top 5%

Cheating in the first block of the Spot-The-Differences task has a positive effect on the probability of cheating in the second block (estimated mean = 1.31, 95% CI = [1.17; 1.45], p-value < .001). The results show a statistically significant effect of the Stress treatment on the probability of cheating (estimated mean = .22, 95% CI = [.07; .38], p-value < .001). In addition, we find support for an interaction effect between stress and baseline cheating (estimated mean = .18, 95% CI = [-.02; .38], p-value = .039). No statistically significant difference was found between the control and stress groups in the percent of trials participants cheated during the first block of the SPOT (*F*(1, 107) = .11, *p*-value = .74). The mean (SD) cheating for the control group (n = 57) is .42 (.23) in the first block and .40 (.30) in the second block. For the stress group (n = 52), the mean (SD) cheating is .43 (.25) in the first block and .46 (.32) in the second block.

Results with a subsample (n = 86) that excludes the bottom 15% and top 15%

Cheating in the first block of the Spot-The-Differences task has a positive effect on the probability of cheating in the second block (estimated mean = 1.12, 95% CI = [.94; 1.29], p-value < .001). The results show a statistically significant effect of the Stress treatment on the probability of cheating (estimated mean = .27, 95% CI = [.11; .43], p-value < .001). In addition, we find support for an interaction effect between stress and baseline cheating (estimated mean = .65, 95% CI = [.37; .93], p-value < .001). No statistically significant difference was found between the control and stress groups in the percent of trials participants cheated during the first block of the SPOT (*F*(1, 84) = .002, *p*-value = .96). The mean (SD) cheating for the control group (n = 46) is .41 (.18) in the first block and .39 (.25) in the second block. For the stress group (n = 40), the mean (SD) cheating is .41 (.18) in the first block and .44 (.30) in the second block.

Results with a subsample (n = 114) that excludes the six participants who removed their hand during the hand immersion task

Cheating in the first block of the Spot-The-Differences task has a positive effect on the probability of cheating in the second block (estimated mean = 1.36, 95% CI = [1.23; 1.50], p-value < .001). The results show a statistically significant effect of the Stress treatment on the probability of cheating (estimated mean = .23, 95% CI = [.08; .39], p-value = .002). In addition, we find support for an interaction effect between stress and baseline cheating (estimated mean = .17, 95% CI = [-.02; .37], p-value = .041). No statistically significant difference was found between the control and stress groups in the percent of trials participants cheated during the first block of the SPOT (F(1, 112) = .002, p-value = .96). The mean (SD) cheating for the control group (n = 60) is .43 (.25) in the first block and .42 (.31) in the second block. For the stress group (n = 54), the mean (SD) cheating is .43 (.28) in the first block and .46 (.35) in the second block.


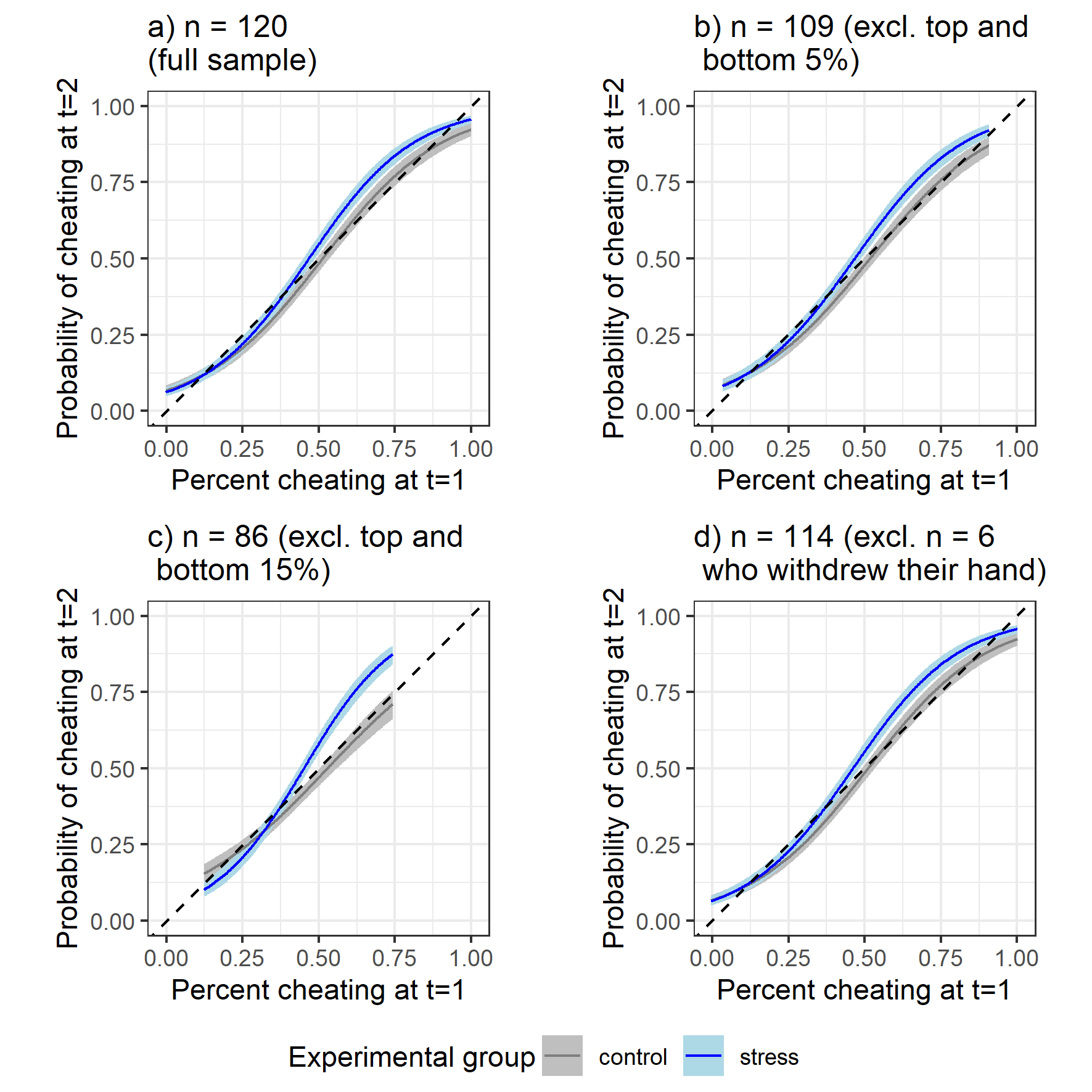


Figure 2: Estimated probability of cheating in the second block of the SPOT (t = 2) as a function of cheating during the first block (t = 1) and experimental group, for four samples with different exclusion criteria. The range of the *x*-axis matches the observed variation in cheating. Confidence bands represent 95% credible intervals (CI).
